# Supplementary material for: Oral health manifestations and the perceived quality of life among Saudi children: a cross-sectional study
Source: PeerJ. 2024 Nov 19;12:e18556. doi: 10.7717/peerj.18556 (PMC11583907; doi:10.7717/peerj.18556)
Supplement: Supplemental Information 2 [file peerj-12-18556-s002.docx]

**Codes with Labels**

1. Do you have children aging between 2 and 11 years old?

1 – Yes

2 – No

1. I know that my participation is voluntary and that I am free to terminate my participation at any time without finishing the survey.

1 – Yes

2 – Yes, I would participate

3 – No

4 – No, I don’t want to participate

1. I agree to participate in this survey.

1 – Yes

2 – No

1. Region of residence
2. South
3. North
4. East
5. West
6. Central region
7. City of residence
8. Abha
9. Dammam
10. Hofuf
11. Jeddah
12. Jubail
13. Makkah
14. Madinah
15. Najran
16. Qatif
17. Riyadh
18. Taif
19. Yanbu
20. Abu Arish
21. Al hasa
22. Al baha
23. Al badayi
24. Aljouf
25. Almhariq
26. Alkhobar
27. Al kharj
28. Al Khafaji
29. Aldayir
30. Aldawadimi
31. Alrassu
32. Alshariea
33. Alzahran
34. Alearida
35. Alghat
36. Alqurayat
37. Al Qasim
38. Alqanfadha
39. Alqawayeia
40. Almujarada
41. Almajmaa
42. Alnueayria
43. Alnamas
44. Alwasit
45. Ayha
46. Badr
47. Burayda
48. Baghdad
49. Baqa
50. Blahmar
51. Bayshin
52. Tabuk
53. Hail
54. Tharmada
55. Jizan
56. Hail
57. Harimala
58. Hafr Albatin
59. Khamis Mushayt
60. Rafha
61. Sabt Alealaya
62. Skakan
63. Sayhat
64. Shuruh
65. Shamal sharq almamlakat hi alkuayt
66. Samitah
67. Sabiana
68. Safwah
69. Arar
70. Asir
71. Eaniza
72. Qaryah / Qaryat Quraish
73. Quisina
74. Sindhi
75. Muhafizuh
76. Mhatan
77. Rhabigh
78. Wadi Aldawasir
79. Maternal education
80. Illiterate
81. Elementary / Middle education
82. High school
83. College or above
84. Paternal education
85. Illiterate
86. Elementary / Middle education
87. High school
88. College or above
89. Maternal education in two groups full sample
90. High school or less
91. University or more
92. Father education in two groups total sample
93. High school or less
94. University or more
95. child age in three groups
96. 2-5
97. 6-8
98. 9-11
99. child order in four groups
100. 1^st^ child
101. 2^nd^ and 3^rd^
102. 4^th^ or more
103. Only child
104. child order in five groups
105. first child
106. second child
107. third child
108. 4^th^ or more
109. Only child
110. Gender
111. Male
112. Female
113. How would you describe the health of your child’s teeth and gums?
114. Excellent
115. Very good
116. Good
117. Bad
118. I don’t know
119. How often did your child have a toothache or feel discomfort due to his/her teeth*?
120. Always
121. Sometimes
122. Rarely
123. Never
124. Have you detected any oral lesion in your child’s mouth*? If yes, what is the lesion?
     1. Pain while eating
     2. Teeth discoloration
     3. Bad odor/ bad breath
     4. Swollen gums / swelling
     5. Abscess cyst
     6. Dry mouth
     7. Itchy gums
     8. Tumor
     9. Difficulty in speaking
     10. Difficulty eating
     11. Ulcer / multiple ulcers
     12. White spots in the mouth or gums
     13. Roughness in the skin of the mouth
     14. Burning sensation
     15. Others
125. Yes
126. No
127. did you visit the dentist last year?
128. No
129. Yes
130. How often did your child go to the dentist during this year ?
131. Once
132. Twice
133. Thrice
134. Four times
135. More than four times
136. Did not visit the dentist during this period
137. If your child visited the dentist, what was the reason for the visit?
138. Tooth ache / pain
139. Tooth decay
140. Check-up
141. Facial abscess / swelling
142. Trauma / broken teeth
143. Ulcers in the mouth
144. Follow-up
145. My child is satisfied with appearance of his teeth
146. Yes
147. No
148. I don’t know
149. child is not satisfied with the appearance of his teeth*
150. Yes
151. No
152. I don’t know
153. Usually, my child avoids smiling in public*
154. Yes
155. No
156. I don’t know
157. Are other children make fun of my child teeth*
158. Yes
159. No
160. I don’t know
161. My child has difficulty biting hard food*
162. Yes
163. No
164. I don’t know
165. My child has difficulty in chewing*
166. Yes
167. No
168. I don’t know
169. if the parents any of the quality questions yes or do not know
170. answered yes or do not know
171. no
172. total of oral health quality of life from 5 to 10_ bad to good
173. How often does your child brush his/her teeth ?
174. Twice or more per day
175. Once a day
176. More than once a week
177. Once a week
178. More than once a month
179. He/she never cleans it
180. does the child brush his teeth?
181. Yes
182. No
183. Does your child use any of the following to clean the teeth?: Toothpaste that contains fluoride
184. Does your child use any of the following to clean the teeth?: Dental floss
185. Does your child use any of the following to clean the teeth?: Toothpick
186. Does your child use any of the following to clean the teeth?: Mouthwash
187. Does your child use any of the following to clean the teeth?: Toothbrush
188. Does your child use any of the following to clean the teeth?: Do not use any brush tool
189. Yes
190. No
191. number of dental visits per year in three groups
192. Once
193. 2 or more
194. No
195. does the child experience dental problem total _of 5 questions
196. How often did your child have toothache or feel discomfort due to his teeth_three groups*
197. Always/sometimes
198. Rare
199. Never
200. How often did your child have toothache or feel discomfort due to his teeth_Two groups* _yes_no
201. Yes
202. No
203. if the child visited the dentist last year starting with yes
